# The correlation between HTA recommendations and reimbursement status of orphan drugs in Europe

**DOI:** 10.1186/s13023-016-0501-4

**Published:** 2016-09-06

**Authors:** Paweł Kawalec, Anna Sagan, Andrzej Pilc

**Affiliations:** 1 Drug Management Department, Institute of Public Health, Faculty of Health Sciences, Jagiellonian University Medical College, ul. Grzegórzecka 20, 31-531 Kraków, Poland; 2 European Observatory on Health Systems and Policies, London, United Kingdom; 3 Institute of Pharmacology Polish Academy of Sciences, Kraków, Poland; 4 London School of Economics, Health and Social Care, London, United Kingdom

**Keywords:** Orphan drugs, Ultra-orphan drugs, Oncology orphan drugs, Drug reimbursement, Reimbursement status, Reimbursement decision, Health technology assessment

## Abstract

**Background:**

The aim of this study was to review and compare types of reimbursement recommendations for orphan drugs issued by eight European health technology assessment (HTA) agencies and the reimbursement status of these drugs in the corresponding countries. Separate calculations were also performed for three sub-groups: ultra-orphan drugs, oncology orphan drugs and other (non-ultra, non-oncology) orphan drugs.

**Results:**

We reviewed drugs authorized by the European Medicine Agency (EMA) between 1 November 2002 and 30 September 2015. Among these, we identified 101 orphan drugs. Seventy-nine of them were assessed by eight European HTA agencies. The average rates of positive, conditional and negative reimbursement recommendations issued by these agencies were 55.7 %, 15.3 % and 29.0 %, respectively. On average, 21.2 % of EMA-authorized orphan drugs were reimbursed in the eight European countries studied: 49.0 % of those with positive, 53.6 % of those with conditional, and 16.0 % of those with negative reimbursement recommendations. In addition, 5.4 % of orphan drugs that had not been assessed by any of the eight HTA agencies were also reimbursed. The shares of oncology, ultra, and other orphan drugs that were assessed by HTA agencies were similar, with the lowest share observed in ultra-orphan drugs (72 %) and the highest in other orphan drugs (80 %). In terms of reimbursement, 20 % of oncology orphan drugs, 25 % of ultra-orphan drugs and 21 % of other orphan drugs were reimbursed.

**Conclusions:**

Reimbursement of orphan drugs does not always correspond to the type of HTA recommendation. While the highest rate of reimbursement is observed (unsurprisingly) among drugs with positive or conditional recommendation, a high rate of reimbursement (11 %) is also observed among ultra-orphan drugs that had never been assessed by any HTA agency.

## Background

While the definition of orphan diseases varies between countries, it is generally accepted that diseases affecting between 1 and 8 persons per 10 000 are regarded as orphan or rare diseases. Within the European Union (EU), orphan conditions are defined by the EMA as life-threatening or chronically debilitating conditions that affect no more than 5 in 10 000 people (which is equivalent to approximately no more than 250 000 in the EU (for each condition)) [1]. Making recommendations on the reimbursement of orphan drugs may be difficult for European Health Technology Assessment (HTA) agencies because of the lack of sufficient clinical and cost data. Prices of orphan drugs are often high, when we compare them with prices of non-orphan drugs, due to small therapy populations. As a result, decisions on the public reimbursement and the number of reimbursed orphan drugs vary between EU member states.

Almost all of the eight European HTA agencies issued in the period of the study (from the beginning of August 2015 till the end of December 2015) three types of recommendations: positive, partially positive (conditional) and negative. The Dutch HTA agency did not issue negative recommendations while the Swedish one did not issue partially positive recommendations. Information on the types of recommendations issued in the eight countries is summarized in Table 1.

**Table 1:** Types of HTA recommendations for orphan drugs issued in the analyzed period

Country	Types of positive recommendations issued	Types of partially positive / conditional recommendations issued	Types of negative recommendations issued
Germany	- Major additional clinical benefit - Significant additional clinical benefit - Marginal additional clinical benefit	- Additional clinical benefit not quantifiable	- No additional clinical benefit - Lower additional clinical benefit
France	- Major improvement of medical benefit - Important improvement of medical benefit - Moderate improvement of medical benefit	- Minor improvement of medical benefit	- No improvement of medical benefit
Netherlands	- Inclusion on List 1B^b^ – non-interchangeable drug with added therapeutic value - Inclusion on List 1B^b^ with financial access arrangement	- Inclusion on List 1A^a^ – interchangeable drug with equivalent therapeutic value	Not issued
Poland	- Major additional clinical benefit - Significant additional clinical benefit - Marginal additional clinical benefit	- Additional clinical benefit not quantifiable - Minor improvement of medical benefit, high price	- Not recommended
Sweden	- Major additional clinical benefit - Significant additional clinical benefit - Marginal additional clinical benefit	Not issued	- No improvement of medical benefit and very high cost
UK-England	- Recommended	- Recommended for restricted use - Recommended for restricted use with Patient Access Scheme	- Not recommended (or not recommended because of no submission)
UK-Wales	- Recommended	- Recommended for restricted use - Recommended for restricted use with Patient Access Scheme	- Not recommended (or not recommended because of no submission)
UK-Scotland	- Recommended - Recommended with Patient Access Scheme	- Recommended for restricted use - Recommended for restricted use with Patient Access Scheme	- Not recommended (or not recommended because of no submission)

*Sources*: Websites of HTA agencies of the eight countries included in the table

*Notes:* ^a^List 1A includes generics, parallel imported medicines and new dosages of medicines that are already included in the reimbursement list. A shortened reimbursement procedure is possible for such drugs, whereby the Ministry of Health, Welfare and Sport decides on the inclusion of the drug in the Medicine Reimbursement System without the input of the Health Care Insurance Board; 90% of the medicines in this category are fully reimbursed. Products in this category are clustered and reimbursed at an average price. ^b^Products which cannot be clustered, but are reimbursed at the market price, are published on List 1B. Conditions for including a medicine in List 1B are based on the assessment of the therapeutic value and cost-effectiveness. If the therapeutic value of the medicine is too low, it will not be eligible for reimbursement. Maximum wholesale prices are the only cap on the reimbursement price

The aim of this study was to evaluate the relationship between the reimbursement recommendations of HTA agencies in eight countries in Europe and the reimbursement status of orphan drugs in these countries, i.e. the accessibility of such drugs for patients. In this study we answered the question if the positive recommendation of a HTA agency translates to the positive reimbursement decision in case of orphan drugs.

The study covers the following countries and HTA agencies: Germany - G-BA (Gemeinsamer Bundesausschuss), France – HAS (Haute Autorité de Santé); the Netherlands – ZIN (Zorginstituut Nederland;), Poland – AOTMiT (Agencja Oceny Technologii Medycznych i Taryfikacji;); Sweden – TLV (Dental and Pharmaceutical Benefits Agency), and three of the four countries of the United Kingdom (England – NICE (National Institute for Health and Care Excellence), Scotland – SMC (Scottish Medicines Consortium), and Wales – AWMSG (All Wales Medicines Strategy Group).

## Methods

The analysis was based on a review of the Orphanet database. During the first stage of this review, we identified all EMA-authorized drugs that were designated as orphan drugs. The review covered drugs authorized between 1 November 2002, which is when the EMA registered the first orphan drug, and 30 September 2015. For all identified orphan drugs, we collected the following information publicly available on chosen agencies` websites in each of the eight countries: (1) was it assessed by the HTA agency? (2) what type of reimbursement recommendation (positive, conditional, or negative; see Table 1) was issued by the HTA agency for this particular drug? and (3) is the drug actually reimbursed? The same data was then collected separately for three sub-groups of orphan drugs: oncology orphan drugs, ultra-orphan drugs, and non-ultra and non-oncology orphan drugs.

Following the National Institute for Health and Care Excellence (NICE) definitions, we have used the following disease prevalence rates for including a drug in our analysis: prevalence of less than 5 per 10 000 for orphan drugs and less than 1 per 50 000 for ultra-orphan drugs [1]. Information on the prevalence of diseases (i.e. classification as an orphan disease) was taken from the Orphanet database and information on the indications for all selected orphan drugs was taken from the EMA’s website (http://www.ema.europa.eu/ema).

## Results

**Fig. 1:**
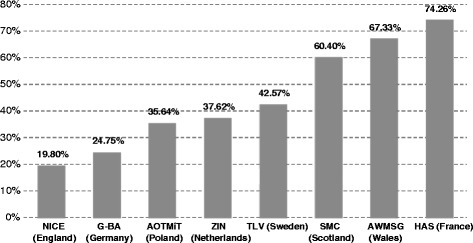
Share (%) of orphan drugs assessed by HTA agency, by country. Source: Authors’ own calculations based on information from the websites of the eight HTA agencies

We have identified 101 EMA-authorized orphan drugs in the period studied. The eight HTA agencies evaluated between 19.8 % and 74.3 % of all identified orphan drugs. The HAS (France) assessed the highest number of orphan drugs (75), while the NICE (England) assessed only 20 (Fig. 1). Among the 101 orphan drugs identified, 22 (22 %) have not been assessed by any of eight HTA agencies.

The average rates of positive, conditional, and negative recommendations issued by the HTA agencies were 55.7 %, 29.0 %, and 15.3 %, respectively. The highest rate of positive recommendations was found in Germany (100 %), and the lowest in the Netherlands (13.2 %). However, the Dutch ZIN agency issued 73.7 % of conditional recommendations, which is the highest rate of conditional recommendations among the eight HTA agencies (Table 2).

**Table 2:** Number and share (%) of assessed and reimbursed orphan drugs by type of HTA recommendation, by country

Country^a^	Assessed orphan drugs (out of 101)		Assessed orphan drugs						Reimbursed orphan drugs										
			Positive		Conditional		Negative		Assessed orphan drugs									Not assessed orphan drugs	
									Total number	Out of 101	Out of assessed drugs	Positive		Conditional		Negative			
England	20 %	20	60 %	12	5 %	1	35 %	7	13	13 %	65 %	25 %	3	0 %	0	14 %	1	11 %	9
Germany	25 %	25	100 %	25	0 %	0	0 %	0	20	20 %	80 %	76 %	19	0 %	0	0 %	0	1 %	1
Poland	36 %	36	19 %	7	36 %	13	44 %	16	23	23 %	64 %	71 %	5	69 %	9	31 %	5	6 %	4
Netherlands	38 %	38	13 %	5	74 %	28	13 %	5	30	30 %	79 %	20 %	1	75 %	21	40 %	2	10 %	6
Sweden	43 %	43	95 %	41	0 %	0	5 %	2	41	41 %	95 %	100 %	41	0 %	0	0 %	0	0 %	0
Scotland	60 %	61	51 %	31	2 %	1	48 %	29	11	11 %	18 %	19 %	6	0 %	0	10 %	3	5 %	2
Wales	67 %	68	28 %	19	9 %	6	63 %	43	13	13 %	19 %	32 %	6	0 %	0	12 %	5	6 %	2
France	74 %	75	85 %	64	9 %	7	5 %	4	20	20 %	27 %	30 %	19	0 %	0	25 %	1	0 %	0

*Source*: Authors’ own calculations based on information from the websites of the eight HTA agencies

*Notes*: ^a^Countries ordered according to the share of assessed orphan drugs (from lowest to highest)

**Fig. 2:**
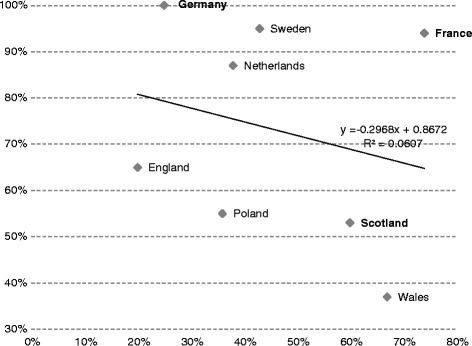
Relationship between the share (%) of assessed orphan drugs (x-axis) and the share (%) of assessed drugs with positive and conditional HTA recommendations (y-axis). Source: Authors’ own calculations based on information from the websites of the eight HTA agencies. Note: Countries where there are special criteria for orphan drugs in the HTA process are marked in bold

Overall, it appears that the higher the number of assessed drugs, the lower the probability of positive and conditional HTA recommendations (Fig. 2).

**Fig. 3:**
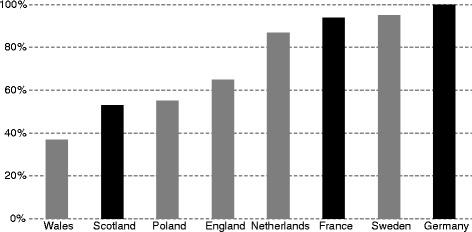
Share (%) of positive and conditional HTA recommendations, by country. Source: Authors based on information from the websites of the eight HTA agencies. Note: Countries where there are special criteria for orphan drugs in the HTA process are marked in black

The analysis does not warrant any statements about causal relationship between the existence of special HTA criteria and the share of positive and/or conditional HTA recommendations or the number of reimbursed orphan drugs. However, out of the three countries that have special HTA criteria for orphan drugs (France, Germany and Scotland), two (France and Germany) have very high rates of positive and conditional HTA recommendations for such drugs (Figs. 2 and 3).

**Fig. 4:**
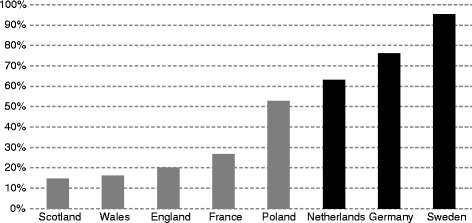
Share (%) of all assessed orphan drugs that are reimbursed, by country. Source: Authors based on information from the websites of the eight HTA agencies. Note: Countries where there are special criteria for orphan drugs in the reimbursement decision process are marked in black

**Fig. 5:**
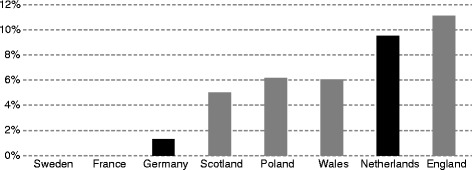
Share (%) of orphan drugs that have never been assessed by any of the HTA Agency considered that are reimbursed, by country. Source: Authors based on information from the websites of the eight HTA agencies. Note: Countries with special criteria for orphan drugs in the reimbursement decision process are marked in black

Countries that have special criteria for orphan drugs in the reimbursement process seem to have higher shares of orphan drugs (as a share of all assessed orphan drugs) that are actually reimbursed – as a share of all assessed orphan drugs (Germany, Netherlands, Sweden; Fig. 4). The existence of special criteria for orphan drugs in the reimbursement process seems to play a lesser role for non-assessed orphan drugs (Fig. 5). The existence of special criteria for orphan drugs in the HTA and reimbursement processes may therefore have some impact on the access to such drugs for patients.

If all positive HTA recommendations translated into actual reimbursement, the Netherlands and Poland would have the lowest number of reimbursed orphan drugs (5 and 7, respectively) and France the highest (64), followed by Sweden (41) and Scotland (31). However, in most countries, the number of reimbursed orphan drugs was much lower than the number of positive HTA recommendations (e.g. 20 vs. 64 for France). In England, Poland and in the Netherlands the number of reimbursed orphan drugs was higher than the number of positive recommendations (Table 2).

On average, 21.2 % of the total of 101 orphan drugs studied (i.e. EMA-approved orphan drugs) were reimbursed in the European countries. The highest rate of reimbursed orphan drugs was observed in Sweden (40.6 %) and the Netherlands (29.7 %). The lowest rate was reported in Scotland (10.9 %). In terms of the share of reimbursed orphan drugs in the total number of assessed orphan drugs, the highest values were observed in Sweden (95 %), Germany (80 %) and the Netherlands (79 %) and the lowest in Scotland (18 %), Wales (19 %) and France (27 %) (Table 2).

The highest rate of reimbursement was observed for drugs that obtained a positive or conditional recommendation from a HTA agency (49.0 % and 53.6 %, respectively (for all countries), compared to 16.0 % for negative recommendations). Poland and the Netherlands were the countries where the highest shares of drugs with a negative recommendation (31.3 % and 40.0 % of drugs, respectively) were reimbursed from public funds. On average, 5.4 % of orphan drugs that have never been assessed by any HTA agency were reimbursed (Table 2).

### Ultra-orphan, oncology, non-ultra and non-oncology orphan drugs

Among the 101 identified orphan drugs, 18 (17.8 %) were classified as ultra-orphan, 34 (33.7 %) were registered with oncologic indications and 50 (49.5 %) were non-ultra and non-oncology orphan drugs. The eight HTA agencies evaluated between 5.6 % (England, Germany) and 72.2 % (Wales, France) of all identified ultra-orphan drugs, between 32.4 % (Germany, Netherlands) and 76.5 % (France) of all identified oncology orphan drugs and between 14.0 % (England) and 74.0 % (France) of all non-ultra and non-oncology orphan drugs. Among the ultra-orphan, the oncology and the non-ultra and non-oncology orphan drugs, 5 (28 %), 7 (21 %) and 10 (20 %), respectively, have never been assessed by any of the eight HTA agencies.

The highest rate of reimbursed drugs with positive HTA recommendations is observed among ultra-orphan drugs (53 %), while among other groups this rate ranges from 46 % (oncology orphan drugs) to 49 % (non-ultra, non-oncology orphan drugs). About 5 % of drugs which have never been assessed by an HTA agency is reimbursed from public funds. This rate is much higher for ultra-orphan drugs - 11 % (Table 3).

**Table 3:** Share (%) of reimbursed ultra-orphan, oncology orphan and other orphan drugs by type of HTA recommendation, by country

	All orphan (101)				Ultra-orphan				Oncology orphan				Non-ultra, non-oncology orphan drugs			
	Positive	Conditional	Negative	Not assessed	Positive	Conditional	Negative	Not assessed	Positive	Conditional	Negative	Not assessed	Positive	Conditional	Negative	Not assessed
England	25 %	0 %	14 %	11 %	0 %	0 %	0 %	24 %	33 %	0 %	0 %	0 %	20 %	0 %	50 %	12 %
Germany	76 %	0 %	0 %	1 %	100 %	0 %	0 %	0 %	73 %	0 %	0 %	0 %	77 %	0 %	0 %	3 %
Poland	71 %	69 %	31 %	6 %	100 %	100 %	50 %	27 %	100 %	67 %	38 %	0 %	33 %	57 %	17 %	3 %
Netherlands	20 %	75 %	40 %	10 %	0 %	75 %	100 %	8 %	0 %	83 %	33 %	17 %	33 %	72 %	0 %	7 %
Sweden	100 %	0 %	0 %	0 %	100 %	0 %	0 %	0 %	100 %	0 %	0 %	0 %	100 %	0 %	0 %	0 %
Scotland	19 %	0 %	10 %	5 %	40 %	0 %	20 %	13 %	9 %	0 %	0 %	8 %	19 %	0 %	14 %	0 %
Wales	32 %	0 %	12 %	6 %	60 %	0 %	0 %	20 %	13 %	0 %	10 %	0 %	29 %	0 %	16 %	7 %
France	30 %	0 %	25 %	0 %	18 %	0 %	0 %	0 %	36 %	0 %	0 %	0 %	35 %	0 %	25 %	0 %
All countries^a^	49 %	54 %	16 %	5 %	53 %	67 %	19 %	11 %	46 %	41 %	14 %	4 %	49 %	57 %	17 %	5 %

*Source*: Authors based on information from the websites of the eight HTA agencies; ^a^ calculated as the sum of all reimbursed drugs with particular type of recommendation (positive/conditional/negative/no recommendation) from all agencies divided by the sum of all drugs (reimbursed and not reimbursed) with particular type of recommendation from all agencies

In terms of access to ultra, oncology, non-ultra and non-oncology orphan drugs, the highest rate of reimbursement is observed in ultra-orphan drugs (25 %). In other groups, i.e. oncology orphan drugs and non-ultra, non-oncology orphan drugs this rate is around 20–21 %. Poland has the highest rate of reimbursement for ultra-orphan drugs (50 %) while Germany has the lowest (6 %). In Sweden, 47 % of oncology-orphan drugs are reimbursed from public funds and only 6 % of such drugs are reimbursed in England, Wales and Scotland. The highest rate of reimbursement for non-ultra, non-oncology orphan drugs is observed in Sweden (36 %) and the lowest in Scotland (10 %).

**Fig. 6:**
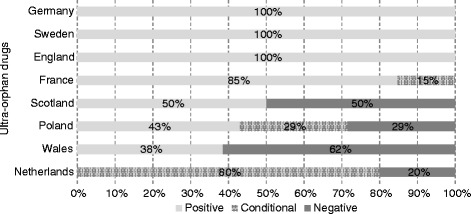
Share (%) of positive, conditional and negative HTA recommendations for ultra-orphan drugs, oncology orphan drugs as well as non-ultra non-oncology drugs; by country. Source: Author’s own calculations based on data from the websites of the eight HTA agencies

The average rates of positive, conditional and negative recommendations issued by all HTA agencies for ultra-orphan drugs were 56.1 %, 15.8 % and 28.1 %, respectively. The highest rate of positive recommendations for ultra-orphan drugs was seen in England, Sweden and Germany (100 %) and the lowest in the Netherlands (0 %); however, the Dutch ZIN issued 80 % of conditional recommendations for ultra-orphan drugs. The average rates of positive, conditional and negative recommendations for oncology orphan drugs were, respectively, 60.2 %, 12.8 % and 27.1 %. The highest rate of positive recommendations for oncology orphan drugs was observed in Sweden and Germany (100 %) and the lowest in the Netherlands (18.18 %). In case of non-ultra and non-oncology orphan drugs the average rates of positive, conditional and negative recommendations were, respectively, 53.3 %, 16.7 % and 30.0 % (Fig. 6). The highest rate of positive recommendations for above group of drugs was observed in Germany (100 %) and the lowest in the Netherlands (13.64 %).

**Fig. 7:**
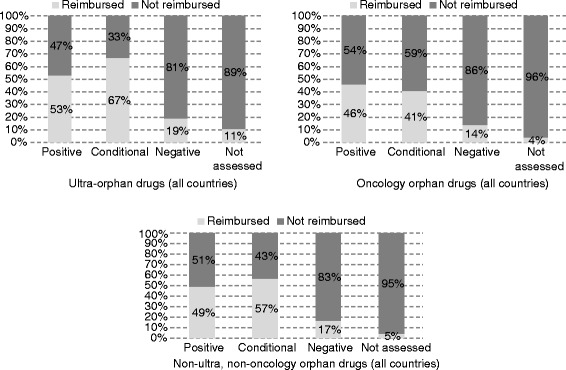
Share (%) of reimbursed ultra-orphan and oncology orphan drugs by type of HTA recommendation, all countries

On average, 25 % of EMA-authorized ultra-orphan drugs, 19.9 % of oncology orphan drugs, 20.8 % of non-ultra and non-oncology orphan drugs were reimbursed in the eight countries. The shares of reimbursed ultra-orphan drugs and non-ultra, non-oncology orphan drugs in the total number of such drugs with positive, conditional and negative reimbursement recommendations (and in the total number of not assessed ultra-orphan drugs) were on average higher than the respective shares for oncology orphan drugs (Fig. 7).

## Discussion

In many European countries HTA is used to assess the value of new technologies, including orphan drugs. This is usually more difficult for orphan compared to non-orphan drugs because reliable clinical and economic evidence required for this purpose is often unavailable for the former due to small numbers of patients. This is despite incentives for pharmaceutical companies to develop such products and the use of less stringent criteria for trials of drugs with designated orphan indications [11].

Based on our research it appears that the higher the number of assessed drugs, the lower the number of positive and conditional HTA recommendations. Some of countries (France, Scotland) apply special criteria to orphan drugs in the HTA process or while deciding on the reimbursement (Netherlands, Sweden) or both (Germany) (Table 4). There seems to be a positive correlation between the existence of such special HTA criteria and the shares of orphan drugs with positive or negative HTA recommendations and between the existence of special criteria in the reimbursement process and the shares of orphan drugs that are actually reimbursed. As it was not an objective of our analysis, we suggest further studies on this topic in a future.

**Table 4:** Special HTA and reimbursement considerations for orphan drugs

Country	Special HTA considerations for orphan drugs	Special reimbursement considerations for orphan drugs
Germany	• Certain special HTA criteria are applied to orphan drugs: - Higher p-values for small sample sizes - Use of surrogate endpoints - Additional benefit is considered proven at marketing authorization (MA) if the budget impact is less than €50 million per year for a particular indication^a^ • Higher therapeutic benefit is automatically recognized for orphan drugs (Section 35a, para. 1 clause 10 of the German Social Code Book V), since these drugs had to prove significant additional therapeutic benefit compared to other possibly already approved drugs as part of the European marketing authorization procedure	• The ascertainment of an additional benefit, which is automatic for orphan drugs, is also binding for subsequent administrative acts, which includes reimbursement decisions by the G-BA (body issuing reimbursement decisions) • IQWiG (body issuing HTA recommendations) only assesses target population size and drug budget impact to all population, and the G-BA decides only on the extent of additional benefit (this applies to all drugs, not only orphan drugs) • While there are no specific pricing considerations for orphan drugs, the latter are often characterized as having no therapeutic alternatives (by G-Ba and IQWiG) - this makes comparison with existing therapies impossible and means free pricing in practice
France	• Certain special HTA criteria are applied to orphan drugs: - Additional benefit is considered proven at MA if the budget impact is less than €30 million per year for a particular indication - Accelerated HTA procedure is available for all innovative drugs (not only for orphan drugs) • The Ministry of Health decides on the reimbursement of the drug, taking into the SMR and ASMR considerations • The Agency for the Sanitary Security of Health Products (Agence Française de Sécurité Sanitaire des Produits de Santé) can issue authorization for temporary use in case of life-threatening conditions or/and when there is no therapeutic alternative (this is not specific to orphan drugs but can be applied to them)	None
Netherlands	None	• Hospitals may apply for full additional funding for orphan drugs that are prescribed within their institution. The additional temporally funding considers therapeutic value, cost prognosis and outcomes research – treatment of all patients need to be documented in a patient registry • In case of orphan drugs the therapeutic value, the severity of the disease and the efficient prescription will be important for the decision on definitive listing/ funding
Poland	None	None
Sweden	None	• TLV (body issuing reimbursement decisions) usually accepts a higher willingness-to-pay threshold for treatment of severe conditions; the human value principle implies equality of all people, while the principles of need and solidarity imply that conditions for which there is a greater need take precedence over others; in practice this means a higher cost-effectiveness threshold may be considered for orphan drugs
England	None	None
Scotland	• Certain special HTA criteria are applied to orphan drugs: - Lower levels of evidence are accepted for clinical trials (e.g., on efficacy and safety) and in economic evaluations - Additional data may be required (e.g., surrogate markers and quality-of-life data)	None
Wales	None	None

*Sources*: [2–10]

*Notes*: ^a^With the exception of orphan drugs, the new Pharmaceutical Market Reorganisation Act of 2010 made the early evaluation of the additional benefit of a pharmaceutical product by the G-BA mandatory after MA; nevertheless, manufacturers of orphan drugs need to submit a dossier so that the G-BA can assess the level of additional benefit and use this in price negotiations, if needed [6]. *MA* marketing authorization

In terms of HTA rejection rates (probability of negative HTA recommendations), Mardiguian et al. [12] found that NICE in England had the highest rejection rate (40 %) while SMC in Scotland had one of the lowest rejection rates (30 %) among the five countries considered (Australia, Canada, England, Scotland and Wales). In our analysis we found that England had a lower rejection rate than Scotland (35 % compared to 47.5 %). This is likely explained by the fact that our sample size (101 drugs) was much higher than that of Mardiguian and colleagues (29 drugs) but comparisons of countries with different healthcare systems (England vs. Australia or Canada) should be performed with caution. Mycka et al. [13] compared orphan drugs assessment in Germany with HTA agencies in five other countries. However, also in this case it is difficult to compare their results with the results of our analysis given the differences in the sample sizes (19 vs. 101) and healthcare systems. To our best knowledge no other evaluations referring to the types of HTA agencies’ recommendations or the relationship between the type of HTA recommendations and the reimbursement status of drugs were performed and published elsewhere

While more than 78 % of approved orphan drugs have been assessed by the European HTA agencies, only a fifth of them (21 %) is reimbursed from public funds. While this also applies to oncology orphan drugs (79 % have been assessed and 20 % have been reimbursed) and non-ultra, non-oncology orphan drugs (80 % and 21 %, respectively), the share of ultra-orphan drugs that are reimbursed is higher (25 %) (72 % of identified ultra-orphan drugs have been assessed by the HTA agencies). This may be because the likelihood of being able to use an alternative treatment for ultra-orphan diseases is much lower than for non-ultra-orphan diseases. Countries that have special criteria for orphan drugs in the reimbursement processes appear to have higher shares of assessed drugs that are reimbursed from public funds; however, this relationship has not been tested in anyway.

It should be noticed that reimbursement recommendations and reimbursement status of drugs are not the same aspects. Due to financial limitations on reimbursement and increasing costs of pharmacotherapy an aggregating difference between positive recommendations and positive reimbursement decisions (what means a real access to pharmacotherapy) is observed in various countries.

Moreover, in majority of European countries no reimbursement is possible for orphan drugs without previous HTA assessment, which is a tool providing decision makers useful information on clinical efficacy, costs and cost-effectiveness of drugs and let allocate public coverage on pharmacotherapy which should be reimbursed in the lack of sufficient financial resources; we speculate that in coming years in all EU member states such assessment for orphan drugs should be obligatory. On the other hand in some countries (e.g. Poland) some changes of too strict reimbursement requirements for orphan drugs are about to be implemented; currently in Poland tjust the same reimbursement procedures as for drugs used in non-rare diseases have been applied for orphans but probably in coming months a new law will be launched in Poland with a submission of a justification of the proposed price of orphan drug instead of submitting a full economic analysis. This means that the cost-effectiveness analyses for orphan drugs will no longer be obligatory but clinical analysis as well as budget impact analysis should still be submitted during application for reimbursement of orphan drugs in Poland. The similar approach to the orphan drugs’ assessment is observed in France – HAS does not examine the economic evidence in a reimbursement process [14]. In Germany there is a lower accepted significance level for p-values in case of assessment of orphan drugs’ clinical outcomes (when sample size is small) and there is an acceptance of evidence from surrogate endpoints. The economic analysis is not required if the budget impact is less than 50 million euro per annum [4]. Lower levels of evidence are accepted for clinical trials for orphan drugs in Scotland and higher cost per QALY than threshold value for non-orphan drugs is accepted in economic analysis. The flexibility in willingness-to-pay threshold value in case of orphan drugs is also acceptable in Sweden [4].

## Conclusions

The reimbursement status does not always correspond to the type of the recommendation issued by an HTA agency for an orphan drug. The highest rate of reimbursement is observed among drugs with positive or conditional recommendation, but high rate of reimbursement is also observed among ultra-orphan drugs that had never been assessed by any HTA agency.

## Abbreviations

AOTMiT: Agencja oceny Technologii Medycznych i Taryfikacji (Polish HTA Agency)
AWMSG: All Wales Medicines Strategy Group
EMA: European Medicines Agency
EU: European Union
G-BA: The General Joint Committee of Germany (Gemeinsamer Bundesausschuss)
HAS: French National Authority for Health (Haute Autorité de Santé)
HTA: Health Technology Assessment
NICE: National Institute for Health and Care Excellence
SMC: Scottish Medicines Consortium
TLV: Dental and Pharmaceutical Benefits Agency (Tandvårds- och läkemedelsförmånsverket)
ZIN: National Health Care Institute (Zorginstituut Nederland)
